# Exploring perspectives on implementing cervical length screening to prevent preterm birth in nulliparous women: protocol for the PRE-EMPT qualitative work packages

**DOI:** 10.1136/bmjopen-2026-121899

**Published:** 2026-10-07

**Authors:** Caroline E Fox, Martina Sykorova, Shazia Zafar, Jenny Carter, Laura Kudrna, Jelena Jankovic, Amy Grove, Charlotte Leeson, Tommer Spence, Magdalena Skrybant, Ellie Jones, Eleanor V Williams, Sarah J Moorlock, Christine MacArthur

**Affiliations:** 1 Birmingham Women’s and Children’s NHS Foundation Trust, Birmingham, UK; 2 Department of Applied Health Sciences, University of Birmingham, Birmingham, UK; 3 Department of Women and Children’s Health, Kings College London, London, UK; 4 Birmingham and Solihull Mental Health NHS Foundation Trust, Birmingham, UK; 5 Centre for Evidence and Implementation Science, University of Birmingham, Birmingham, UK; 6 Health Services Mangement Centre, University of Birmingham, Birmingham, UK

**Keywords:** Pregnancy, Mass Screening, OBSTETRICS

## Abstract

**Abstract:**

**Introduction:**

Preterm birth risk prediction currently relies on previous history, with those classified as intermediate-risk or high-risk being offered cervical length measurements. However, most preterm births occur in women considered low-risk. The majority of low-risk women are first-time mothers (nulliparous women) and therefore, typically do not receive cervical length measurements. Evidence from outside the UK suggests that cervical length screening and treatment with progesterone in low-risk women can prevent preterm birth. However, little is known about the acceptability and feasibility of introducing cervical length screening in low-risk nulliparous women in the UK National Health Service (NHS). This study aims to provide this evidence to inform decision-making for cervical length screening (NIHR PGfAR 207290).

**Methods and analysis:**

A study consisting of two theoretically informed qualitative work packages will explore the perspectives and experiences of women, healthcare professionals and other relevant stakeholders regarding cervical length screening for low-risk nulliparous women. Data will be collected through a combination of semistructured interviews and focus groups with approximately 50 healthcare professionals and 50 women and an online survey.

Women will be purposively sampled from community groups and maternity units. Healthcare professionals and providers will be identified through purposive and snowball sampling from maternity units and healthcare and other relevant organisations including those involved in the planning and commissioning of antenatal services. All interviews and focus groups will be audio-recorded and transcribed verbatim. Posters advertising the study will be displayed in maternity units and community centres. Data collection began in September 2025 and is due to end in December 2026. Data will first be analysed inductively and then deductively using the Behaviour Change Wheel, Theoretical Domains Framework and the Feasibility Framework.

**Ethics and dissemination:**

Ethical approval was granted by the Health Research Authority (IRAS 349196) and NHS Research Ethics Committee (25/NW/0252) as well as the University of Birmingham (RG_25-018). Findings will be shared with service users and healthcare providers through peer-reviewed publications and conferences. Results will also be reported to the UK National Screening Committee to inform recommendations on national adoption.

**STRENGTHS AND LIMITATIONS OF THIS STUDY**

- Theoretically informed insights about behavioural, feasibility and implementation drivers, processes and strategies for change.
- Comprehensive examination of multi-level factors affecting the implementation of a cervical length screening pathway for low-risk nulliparous women, incorporating the perspectives of diverse stakeholders across the UK.
- Data collection will be largely virtual, which may constrain the researcher’s ability to establish rapport with participants, although it does offer more flexibility and may attract potential participants who would otherwise decline participation.
- No causal evaluation of the effectiveness of behavioural and feasibility and implementation strategies and approaches to reducing preterm birth will be conducted, limiting our understanding of its benefits and risks in this context.

## Introduction

 Preterm birth (PTB), defined as birth before 37 weeks’ gestation, is the leading cause of perinatal morbidity and mortality in high-income countries^1^ and remains the foremost cause of death among children under 5 globally.^2^ In the UK, approximately 1 in 12 babies are born prematurely every year.^3^ In 2023, 8.1% of all births in England occurred between 24 and 36 weeks’ gestation, a rate slightly below the global average of approximately 10%.^4^ The burden of PTB is not evenly distributed, with markedly poorer outcomes observed among Black, Asian and socioeconomically deprived populations.^3 5^ In response, the UK Department of Health and Social Care set a target to reduce the national PTB rate from 8% to 6% by 2025; however, this target had not been achieved by 2026.^6^ Reflecting ongoing concerns regarding the persistent prevalence of PTB, associated health inequalities and limited progress in prevention, the UK government established a Lords Select Committee on Preterm Birth in January 2024 to examine strategies for prevention and mitigation of its consequences.^7^

Most PTBs occur in women defined as low-risk, many of whom are first-time mothers (nulliparous women).^8 9^ Cervical length (CL) measurement is an established predictor of PTB as a short cervix is associated with increased risk^10^ and can be treated with progesterone.^11^ In the UK, CL scans and progesterone are currently offered to women at high-risk or intermediate-risk of PTB. Higher risk women include those with a history of prior preterm delivery, mid-trimester loss, cervical surgery or uterine congenital anomaly.^12 13^ However, there is some evidence that offering CL measurements and associated progesterone treatment reduces PTBs in lower risk populations. For example, a 2013 Cochrane review showed that screening an unselected population for short CL reduces the risk of PTB prior to 34 weeks (risk ratio 0.64, 95% CI 0.45 to 0.90).^14^ In addition, a recent meta-analysis suggests that screening women without a previous PTB is associated with a significant reduction in spontaneous PTB, compared with no screening.^15^ Moreover, economic evidence indicates that universal transvaginal CL screening can be cost-effective in low-risk pregnancies.^16^ Although not part of routine care in the UK, the International Society of Ultrasound in Obstetrics and Gynaecology (ISUOG) recommends universal CL screening in all singleton pregnancies between 18 and 24 weeks’ gestation.^17^ Much of the supporting evidence not included in the 2013 Cochrane review comes from primary studies in France and the USA.^18–22^ Reflecting this evolving evidence base, clinical guidelines remain inconsistent in their conclusions regarding the benefits of universal CL screening as demonstrated by a recent review of guidelines due to the perceived limited accuracy of CL to predict PTB.^23^

PREventing PretErM Birth in nulliParous women Through cervical length screening (PRE-EMPT) (NIHR 207290) is a research programme to assess the feasibility of designing and implementing a screen and treat pathway for nulliparous women in the National Health Service (NHS). The PRE-EMPT study consists of five work packages and is a multicentre UK-wide study (see figure 1). The first two work packages are outlined in this protocol and will inform the design and conduct of the subsequent work packages. Both work packages employ a qualitative approach with interviews and focus groups to explore the perspectives and experiences of healthcare professionals and providers regarding the proposed screening pathway, as well as women who have been offered CL scanning and progesterone treatment or are in their first pregnancy. These studies are conducted in anticipation of potential policy developments in PTB prevention, to generate early evidence on how a screening and treatment pathway could be implemented in practice. Other work packages, described elsewhere, investigate health economic aspects of offering CL screening and treatment and wider adoption. Overall, the approach described in what follows will identify and help suggest feasibility and implementation solutions and behaviour change techniques that can be integrated into a UK screening and treatment pathway.

**Figure 1:**
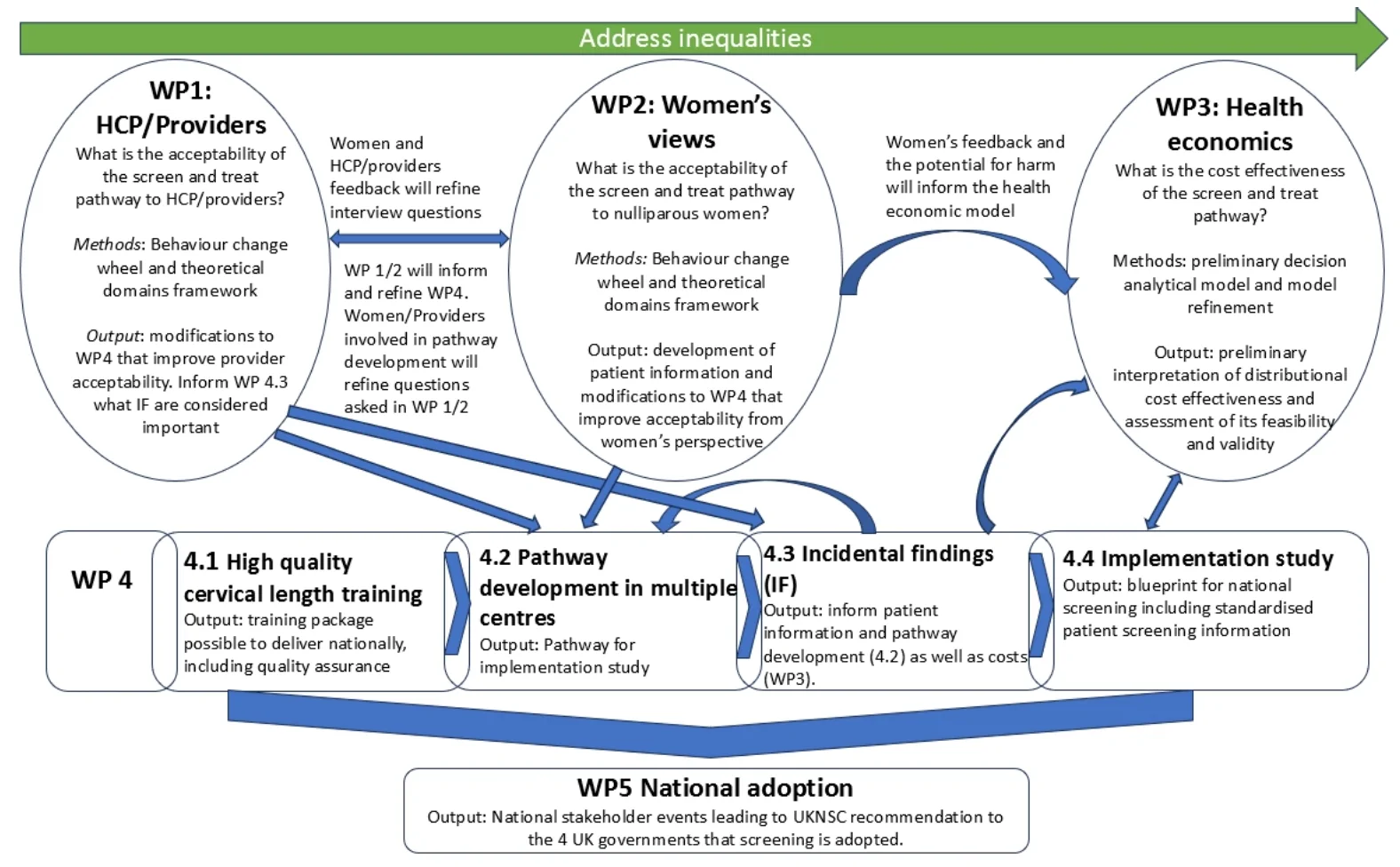
PREventing PretErM Birth in nulliParous women Through cervical length screening (PRE-EMPT) study flow chart demonstrating linkage between work packages. HCP, healthcare professional; UKNSC, UK National Screening Committee; WP1, Work Package 1.

## Methods

### Study aims and objectives

1. Evaluate the acceptability of the screening pathway to healthcare professionals and providers.
2. Explore the barriers and facilitators to accepting CL screening, treatment with progesterone and the psychological impact of the process.

### Research questions

1. What are the views and perspectives of healthcare and other professionals on the acceptability and feasibility of a CL screening and treatment pathway? Should this be offered universally to first-time expectant mothers?
2. What barriers and facilitators do women describe as affecting their uptake of (a) CL scanning/screening and (b) progesterone treatment when offered?
3. What are the perceived psychological impacts of (a) CL scanning/screening and (b) progesterone treatment when offered?

### Study design

A multicentre qualitative study conducted in the UK comprising two parts of a larger programme. Figure 1 depicts the individual work packages and their interactions. Work package 1 (WP1) (research question 1) will include interviews and focus groups with healthcare professionals and providers involved in CL scanning and PTB prevention. WP2 (research questions 2–3) will run concurrently and will consist of interviews with women who have experienced, or could experience, transvaginal ultrasound measurement of CL and/or progesterone treatment. Together, WP1 and WP2 will inform an understanding of the barriers and facilitators to uptake of the screening and treatment pathway.

WPs 1 and 2 will mutually inform one another. The wider PRE-EMPT programme consists of five WPs and this paper focuses on the first two. Protocols for the other work packages will be published separately. Furthermore, insights from WP1 and WP2 will inform WPs 3 and 4. WP 3 will comprise the evaluation of health economic aspects of offering CL screening and treatment. WP 4 will involve the implementation of a screen-and-treat pathway across 12–15 units in the UK to assess whether CL screening can be delivered; refine a screening, treatment, and follow-up pathway and evaluate incidental findings. All WPs will feed into WP 5, which will provide recommendations for national adoption of the CL screening and treatment pathway in the UK, should findings from WPs 1–4 show that implementation is feasible.

### Study setting

Up to 50 healthcare professionals will be recruited for the first qualitative WP1. Broad representation will be sought from maternity units across the UK, as well as organisations involved in PTB prevention and perinatal care, for example, UK Preterm Clinical Network, the British Medical Ultrasound Society and the Society of Radiographers. We will gather their perspectives on various components of the proposed screening pathway, including planning, commissioning, delivery and operational management. Recruitment of up to 50 women to the second qualitative WP2 will be from maternity units and community groups across the UK and for the latter, focusing on community groups that can support access to underserved groups.

### Sample size

The expected sample size is 40–50 healthcare professionals for WP1 and 40–50 women for WP2, across interviews and focus groups. This estimate is informed by previous studies, which conducted between 17 and 89 individual and group interviews,^24–27^ as well as methodological guidance indicating that a minimum of 12 interviews is typically required to reach data saturation.^28^ The final number will be determined using information power and saturation principles,^29 30^ stopping when there is little or no new information coming from data collected during analysis. We will use quota sampling to ensure diverse participation across both work packages. As part of WP2, we will co-develop the sampling quotas with our public contributor group, with consideration given to ethnicity, socioeconomic status, gender identity, geography, culture and religion.

### Eligibility criteria

WP1:

- Professionals involved in PTB prevention such as sonographers, doctors, midwives, commissioners and staff who organise ultrasound services.
- Professionals practising in the UK.

WP2:

- Women who have been pregnant, are pregnant or are expecting to become pregnant in the future.
- Women living in the UK.

### Identification of participants and recruitment

WP1: Healthcare and related professionals will be recruited through professional networks, relevant groups, established research sites as well as participating hospital sites. A short survey, accessible via a quick response (QR) code or web link, will enable potential participants to register their interest. Alternatively, individuals may express interest by contacting the research team directly via email. In addition to purposive sampling, recruitment will also take place through snowball sampling.

WP2: Two recruitment approaches will be used: recruitment through community groups and through maternity units. First, the researcher will collaborate with community organisations to identify eligible participants. Recruitment will be undertaken through attendance at community events, working in collaboration with organisations with established relationships with the communities they serve, including charities, voluntary and community sector organisations, maternity support groups, faith-based organisations and community advocates. This aims to improve recruitment from communities where trust in healthcare organisations or research may be a barrier to participation. Dissemination of recruitment materials will be facilitated via these organisations. Interested individuals will be invited to complete an online form collecting contact details, experiences of transvaginal scans and pregnancy care, and demographic characteristics to enable purposive sampling. Second, eligible participants will be identified and initially approached by their usual care team within maternity units. The research team will then contact individuals who express interest. Women with prior experience of CL scans will primarily be recruited through PTB prevention clinics, where such scans are routinely undertaken among women at high risk of PTB. To ensure a broader understanding of the acceptability of a potential screening programme, we will also interview low-risk nulliparous women attending their mid-trimester fetal anomaly scan regarding their views on CL screening. Including women with and without direct experience of CL scans will enable exploration of a diverse range of perspectives and potential barriers and facilitators to screening uptake.

Recruitment materials will also be displayed in varied healthcare settings, providing study information and enabling self-enrolment via QR codes. As an alternative approach, healthcare professionals may complete a consent-to-contact form for suitable participants, including those who are digitally excluded, and forward this to the research team. For potential participants who are unable to speak English, communication will be facilitated via a translator, community leaders or the researcher (SZ), who is multilingual. Preinterview eligibility checks may be conducted to ensure participants are genuine.

The research team will monitor recruitment across demographic groups throughout data collection and adapt recruitment strategies where under-representation is identified to maximise diversity and inclusion.

A £15 honorarium, provided in the form of a voucher, will be offered to all eligible participants taking part in the interviews or focus groups for WP1 and WP2.

### Data collection

Interviews and focus groups are particularly appropriate for an in-depth examination of participants’ views, attitudes and beliefs^31^ around PTB and CL screening. Both individual interviews and focus groups will be used to provide complementary insights. Interviews will enable detailed exploration of participants’ personal experiences, views and behaviours, particularly where topics may be sensitive or context-specific. Focus groups will allow participants to interact with one another, generating discussion that could reveal shared understandings, diverse perspectives and areas of agreement or disagreement that may not emerge during one-to-one interviews. The use of both methods will facilitate a comprehensive understanding of the study topic and enhance the richness of the dataset through methodological diversification.

Qualitative interviews with individuals and focus groups will explore professionals’ and women’s experiences and perspectives on CL scanning and progesterone treatment, along with the perceived psychological impact. Interviews will be guided by semistructured topic guides (see online supplemental material) which, alongside the participant questionnaire, will be developed by the research team based on the study objectives and a review of the relevant literature. The guides will be designed to explore participants’ experiences, perceptions and priorities relating to CL screening while allowing flexibility to pursue issues raised by participants. Open-ended questions and prompts will be included to facilitate in-depth discussion. Guides will be reviewed by co-applicants, and WP2 guides by public contributors, to establish content relevance, comprehensiveness and clarity. Pilot interviews will be conducted with representatives from the relevant participant populations to assess question clarity, acceptability, interview flow and estimated duration. Feedback from these pilot interviews will be reviewed by the research team to refine the topic guides before formal recruitment begins. The topic guides will be reviewed iteratively throughout data collection and refined where necessary to explore emerging themes. Written informed consent will be obtained prior to each interview or focus group. Participants’ anonymity and confidentiality will be maintained throughout the study. Survey information will be stored on a secure online platform provided by the University of Birmingham and only accessed by appropriate members of the research team. Interviews will be recorded using an encrypted voice recorder. Data will be uploaded into a password-protected university OneDrive folder which will be only accessible to the members of the research team. Recordings will be deleted from the portable voice recorder as soon as they are uploaded to the university drive. Data will be transcribed by a professional transcription company and anonymised immediately on transcription. Audio recordings will be deleted once transcribed and checked. Participants will only be identifiable using a study identification number. Electronically signed consent forms will be digitalised and uploaded on the university password-protected drive. Physical copies of the consent forms will then be destroyed. In-person signed consent forms will be stored securely in a locked unit at the University of Birmingham. Access to consent forms will be restricted to authorised members of the research team, in accordance with ethical approval requirements and the UK General Data Protection Regulation.

WP1 will use tailored topic guides for different participant groups, that is, healthcare professionals, those in strategic roles and commissioners to reflect their distinct roles and experiences. These topic guides will be informed by constructs from implementation science frameworks, ensuring exploration of factors critical to implementation success and feasibility.

WP2 will use topic guides tailored to the individual (either in the community or maternity unit) with questions to explore their experience of CL scanning and/or treatment if they have had it, or discuss what they think about having these if they have not. The topic guides will be informed by the Behaviour Change Wheel, which identifies participants’ capabilities, opportunities and motivations to perform a behaviour.^32^

Topic guides for both work packages will be reviewed periodically and revised according to emerging themes and feedback from participants. The interviews and focus groups will be conducted by experienced post-doctoral qualitative researchers. The WP1 interviews will be conducted by MSy and TS and the WP2 interviews by SZ. All are applied health researchers with expertise in qualitative methods and maternity care research. They have no prior relationships with participants and, through regular discussions with the wider research team, will continuously reflect on the impact of their professional backgrounds, assumptions, interpretations and prior knowledge on the data collected. They will also consider how their positionality may influence participants’ willingness to share their experiences and consider how to further support the inclusion of underserved groups and perspectives that may otherwise be overlooked.

Interviews and focus groups will last approximately 60 min and will be conducted at a time and place convenient for participants. Most interviews and focus groups will be conducted online (via Microsoft Teams/Zoom) but may be face-to-face depending on the preferences of the participant. Participants will be encouraged to keep their camera on to allow for the observation of body language as well as to ensure participants are genuine respondents. Conducting interviews online will allow for better access to some underserved participants or overcome geographical constraints.^31^

### Data analysis

The analysis will start with familiarisation of the data, where interview and focus group transcripts will be read several times.^33^ Data will be managed using NVivo (V.1.7.1; QSR International). Coding frameworks for each work package will be developed iteratively as analysis progresses. First, an inductive coding approach will be applied to transcripts. Second, constructs from behavioural and implementation science frameworks will be applied to the data to elucidate further insights that are grounded in theory and existing evidence. This approach supports theory development to inform the implementation of healthcare interventions, such as the screening and treatment pathway, into routine practice.^34^ In WP1, feasibility and implementation insights will be mapped onto the Feasibility Framework.^35^ This framework examines multiple dimensions of feasibility, including acceptability, practicability and implementation, to assess the conditions under which the pathways can be successfully implemented and evaluated. In WP2, behavioural insights will be mapped onto the Behaviour Change Wheel and Theoretical Domains Framework.^32^ Overall, the identified barriers, facilitators, behaviour change techniques and feasibility and implementation strategies from both work packages will be integrated to inform the design of the screening and treatment pathway for the subsequent implementation study (WP4).

WP1 analysis will be performed by MSy and CL. WP2 analysis will be performed by SZ and CL.

### Analytical rigour

To support credible interpretations of the data, approximately 10% of transcripts will be independently coded by another member of the research team from a different research background (eg, clinical maternity or methodological). Comparison of assigned codes will be followed by a discussion until full code descriptions are achieved. Codebooks will be iteratively refined to exclude or update unclear codes. In addition, interim findings and analysis will also be discussed with the wider research team at fortnightly meetings. A subset of the data will also be double-coded by the same researcher at different time points to assess consistency. Any discrepancies between coding iterations will be examined and reflected on to refine the coding framework. An audit trail will be maintained throughout the process, alongside ongoing reflexive practice. These qualitative work packages will be reported according to the Consolidated criteria for Reporting Qualitative research (COREQ) guideline.^36^

The project protocol has been registered with the Open Science Framework public registry (osf.io/fs7tc).

### Public and patient involvement

Public involvement in the PRE-EMPT project is co-led by an experienced Public Involvement Lead (MSk) working in partnership with a public contributor with lived experience of PTB. The initial study design for PRE-EMPT, including the design of a detailed public involvement plan, was informed by two online and face-to-face discussion sessions in March and August 2023 led by CEF, MSk and EJ. Women with lived experience of PTB were recruited from charity groups and partners, specifically Tommy’s, Sands and Approachable Parenting, while first-time mothers were recruited from a Parentcraft group in Birmingham.

Once funding was awarded, a public and patient involvement (PPI) group of 10 women was established and the approach to PPI aligns to the UK Standards for Involvement.^37^ PPI members of the group, who named themselves the Women’s Advisory Group (WAG), come from a range of cultural and socioeconomic backgrounds. They also contribute insights from different experiences during pregnancy, including experience of CL scan and progesterone treatment, PTB and pregnancy loss. While most public contributors recruited had no prior experience of public involvement, two members had significant experience of public involvement in maternity-related projects. All contributors attended a project induction, with introductions to the project team.

WAG meetings are hybrid (online and face-to-face), ensuring accessible opportunities for involvement and are held at least four times a year, with additional meetings arranged if required. In addition to providing regular updates on project progress, WAG meetings will provide opportunities for members to contribute to all aspects of study design and delivery. For WP2, WAG input will inform the design and content of all participant-facing information, including recruitment posters, participant information sheets and consent forms, ensuring content, layout and style are clear and accessible. WAG members’ input will also inform the design and content of interview topic guides, ensuring issues important to women are incorporated into questions, and for WP2 in particular, ensuring that the framing of the questions, language and style used are appropriate, clear and sensitive. WAG members will also have an important role in shaping how findings from both work packages can be shared, including identifying suitable audiences and working with the wider team to use the findings to inform work packages on health economics (WP 3) and the screening and treatment pathway (WP 4).

Feedback continues to be provided to all WAG members on how their contributions make a difference to the project, and PPI is reported using the Guidance for Reporting Involvement of Patients and the Public(GRIPP2) Short Form.^38^

### Project governance

The study is overseen by a Programme Steering Committee composed of senior clinicians, researchers, our public collaborator and academics who advise on the study design, protocol, and any amendments, as well as providing guidance on issues that arise during the study process and routes for implementation.

### Study timeline

Participant recruitment and data collection started in September 2025 and is expected to last until December 2026, until fully recruited or until data saturation is achieved.

At the time of manuscript submission, recruitment and data collection are ongoing and semistructured interviews are being conducted. No formal analysis has yet been undertaken. Following completion of data collection, interview recordings will be transcribed and analysed according to the methods outlined in this protocol.

## Discussion

The PRE-EMPT programme aims to explore the feasibility and acceptability of a universal CL screening and treatment pathway, the latter with the use of progesterone. Vaginal progesterone has been shown to reduce PTB in women with singleton pregnancies and a short cervix,^39 40^ with no long-term harms observed in children at 2 years of age.^41^ Taken together, the PRE-EMPT programme has the potential to deliver meaningful benefits for women, their families and the NHS. We acknowledge that the success of this routine screening is dependent on acceptability of women, their families and the healthcare systems.

Considering ongoing national efforts to strengthen maternal healthcare provision in the UK, there is an increasing need to identify and implement projects that meaningfully improve the quality and accessibility of these services. The drive to reduce premature birth is reflected in the substantial growth of PTB services across the UK, increasing by 160% in England over 4 years.^42^ Unfortunately, this has not led to an associated reduction in PTB, with rates increasing in all demographic groups and more so in ethnic minority and socioeconomically deprived women.^43^

Extending CL screening to all first-time mothers, rather than limiting it to those with pregnancies at high-risk or intermediate-risk of PTB, could enable earlier identification of women who may benefit from treatment with progesterone and thereby support safer pregnancies.^44^ Evidence from the USA further suggests that universal CL screening can result in healthcare cost savings and gains in quality-adjusted life-years.^16^ However, despite these potential benefits, there is limited evidence regarding the acceptability and feasibility of universal CL screening within the UK healthcare context, particularly among low-risk nulliparous women.

This study is designed to address this evidence gap by exploring the acceptability and feasibility of introducing CL screening for low-risk nulliparous women in the UK. Through in-depth qualitative research to inform the development of a CL screening pathway, we aim to maximise its potential for successful implementation. By incorporating the perspectives of healthcare professionals alongside those of a diverse group of women, the study will help ensure that, when the screening pathway is offered universally in WP 4, it is designed in ways that are feasible, acceptable, and responsive to the needs of all women.

A key strength of this study is its theoretically informed approach which will generate insights into the feasibility of the screening pathway as well as implementation determinants and potential strategies for change. Another strength is the planned involvement of a wide breadth of participants to gain insights from a diverse range of perspectives. A limitation of this qualitative approach is that the research is not designed to evaluate the causal effectiveness of different strategies for reducing PTB but will feed into the implementation study in WP 4 that will collect data to this end. Collectively, the work packages aim to provide rich information to inform the design and implementation of a CL screening pathway that is functional and implementable within the NHS.

## Ethics and dissemination

### Ethical considerations

The study gained approval from the Health Research Authority (IRAS 349196) and NHS Research Ethics Committee (25/NW/0252) as well as the University of Birmingham Research Ethics Committee (RG_25-018). Data protection will be subject to General Data Protection Regulation and UK Data Protection Act 1998.

### Output and dissemination

Findings from the two qualitative work packages (healthcare professional and patient respondents) will be used to inform the health economics evaluation of offering CL screening and treatment (WP 3) as well as development of the screening and treatment pathway which will be evaluated in later stages of the research programme (WP 4, see figure 1). The findings aim to directly influence the design and content of participant information provided during the CL screening implementation (WP 4) as well as the training of sonographers involved in the pathway. Further, key findings from both qualitative work packages will be shared with service users and healthcare providers via lay reports, peer-reviewed publications and conferences. They will also be provided to the UK National Screening Committee for their assessment for recommendation for national adoption. Final study outputs will be communicated to the Department of Health and Social Care.

## Supplementary material

10.1136/bmjopen-2026-121899 online supplemental file 1
