# Supplementary material for: Exploring perspectives on implementing cervical length screening to prevent preterm birth in nulliparous women: protocol for the PRE-EMPT qualitative work packages
Source: BMJ Open. 2026 Oct 7;16(10):e121899. doi: 10.1136/bmjopen-2026-121899 (PMC13646949; doi:10.1136/bmjopen-2026-121899)
Supplement: Supplementary file 1 [file bmjopen-2026-121899.reviewer_comments.pdf]

PEER REVIEW HISTORY

BMJ Open publishes all reviews undertaken for accepted manuscripts. Reviewers are asked to complete a checklist review form and are provided with free text boxes to elaborate on their assessment. These free text comments are reproduced below.

ARTICLE DETAILS

Title (Provisional)

Exploring perspectives on implementing cervical length screening to prevent preterm birth in nulliparous women: protocol for the PRE-EMPT qualitative studies

Authors

Fox, Caroline E.; Sykorova, Martina; Zafar, Shazia; Carter, Jenny; Kudrna, Laura; Jankovic, Jelena; Grove, Amy; Leeson, Charlotte R.; Spence, Tommer; Skrybant, Magdalena; Jones, Ellie; Williams, Eleanor V.; Moorlock, Sarah J.; MacArthur, Christine

---

VERSION 1 - REVIEW

**Reviewer**	**1**
**Name**	**Black, Mairead**
**Affiliation**	**University of Aberdeen**
**Date**	**10-Jul-2026**
**COI**	**none**

This detailed protocol describes two components of a wider programme of work. The protocol is well written and comprehensive.

Minor aspects to consider and clarify in order to improve readability are:

What are the planned study data collection dates? These are essential in order to ensure that criteria for publication of the protocol are met.

Why ‘two theoretically informed studies’ when no further information is provided to differentiate between the two studies? Similarly, the description of ‘This study’ goes on to describe ‘two work package studies’ but doesn’t clearly state which is which. Consider describing the whole study as one (made up of two work packages – stating what each does separately), especially as only one set of research objectives are provided.

Provide justification for using both interviews and focus groups.

Why would low-risk women be attending a preterm birth prevention clinic?

Ensure detailed description of sampling underserved groups, bearing in mind that an online survey is highly unlikely to succeed in recruiting women from underserved groups where trust in authorities is low (applicable to multiple different groups in society). It would also be helpful to highlight any team member characteristics which demonstrate representation of underserved groups. Given the need for substantial trust between research teams and underserved participants, the plans would benefit from more detail than the current intention to recruit via community groups who 'will facilitate access to underserved groups' as access alone does not mean successful recruitment will follow.

In ‘Study 2’ what aspects of topic guides might changes when tailored to community groups or maternity units?

VERSION 1 - AUTHOR RESPONSE

Reviewer comment 1: This detailed protocol describes two components of a wider programme of work. The protocol is well written and comprehensive.
Minor aspects to consider and clarify in order to improve readability are:

Response: We thank the reviewer for the positive assessment of the manuscript and appreciate the encouraging feedback.

Reviewer comment 2: What are the planned study data collection dates? These are essential in order to ensure that criteria for publication of the protocol are met.

Response: Thank you for suggesting this clarification. Please see the main body of the text under ‘study timeline’ in the methods section (page 9, line 363).

Reviewer comment 3: Why ‘two theoretically informed studies’ when no further information is provided to differentiate between the two studies? Similarly, the description of ‘This study’ goes on to describe ‘two work package studies’ but doesn’t clearly state which is which. Consider describing the whole study as one (made up of two work packages – stating what each does separately), especially as only one set of research objectives are provided.

Response: We appreciate this advice to improve clarity. The manuscript has now been edited to describe two work packages that make up a single study.

Reviewer comment 4: Provide justification for using both interviews and focus groups.

Response: Thank you for highlighting the importance of justifying the use of both qualitative data collection methods, we have addressed this in the methods section of the manuscript (page 6, line 222).

Reviewer comment 5: Why would low-risk women be attending a preterm birth prevention clinic?

Response: We appreciate that the approach to patient recruitment could be better explained, thank you for suggesting clarification. This has been edited on page 5, line 198.

Reviewer comment 6: Ensure detailed description of sampling underserved groups, bearing in mind that an online survey is highly unlikely to succeed in recruiting women from underserved groups where trust in authorities is low (applicable to multiple different groups in society). It would also be helpful to highlight any team member characteristics which demonstrate representation of underserved groups. Given the need for substantial trust between research teams and underserved participants, the plans would benefit from more detail than the current intention to recruit via community groups who 'will facilitate access to underserved groups' as access alone does not mean successful recruitment will follow.

Response: We thank the reviewer for this important observation and agree that recruitment of women from underserved groups requires approaches that extend beyond access through community groups. Recruitment will include purposive sampling through established, trusted community networks rather than online survey completion alone. We recognise that the online survey is unlikely to be acceptable to women from communities where trust in healthcare organisations or research may be limited. We also want to avoid assuming that access alone will result in participation.

We have worked alongside our public involvement co-applicant to utilise existing relationships with community organisations that work with women who might not typically engage with research projects due to lack of trust. Recruitment will be undertaken in collaboration with organisations that have longstanding relationships with the communities they serve, including charities, voluntary and community sector organisations, maternity support groups, faith-based organisations and community advocates. These organisations will not simply advertise the study but will act as trusted intermediaries, introducing the research to potential participants, explaining its

purpose, and supporting women to engage if they wish. This has been clarified under the section 'Identification of participants and recruitment' on page 5, line 189.

Reviewer comment 7: In 'Study 2' what aspects of topic guides might changes when tailored to community groups or maternity units?

Response: Thank you for asking for clarification on this point. We should have written that the topic guide will be tailored to the individual, based on what prior experience they have had of cervical length scanning, and developed iteratively throughout the process. We have explained this on page 7, line 266.

VERSION 2 - REVIEW

**Reviewer**	**1**
**Name**	**Black, Mairead**
**Affiliation**	**University of Aberdeen**
**Date**	**23-Aug-2026**
**COI**	

The very first sentence in the abstract would benefit from adding the word 'consisting' before of two work packages, as otherwise it read as it the work packages are being studied.

The new text describing the development of the interview guides is mostly written in the past tense. I would suggest changing to future tense to be consistent with the rest of the paper.

Please include details of study registration on a public register.

VERSION 2 - AUTHOR RESPONSE

Response to the Reviewer Comments

The line numbers referenced below correspond to the tracked changes version of the revised manuscript (Main document – marked copy).

Reviewer comment 1: The very first sentence in the abstract would benefit from adding the word 'consisting' before of two work packages, as otherwise it read as it the work packages are being studied.

Response: Thank you for this improvement. We have added this to the manuscript (page 1, line 18)

Reviewer comment 2: The new text describing the development of the interview guides is mostly written in the past tense. I would suggest changing to future tense to be consistent with the rest of the paper.

Response: Thank you, we have made this change to the manuscript (page 6, line 229 onwards and page 8, line 335 onwards).

Reviewer comment 3: Please include details of study registration on a public register.

Response: Thank you for this suggestion. We have now registered the protocol on Open Science Framework, this can be found at osf.io/fs7tc (see page 8, line 314). We acknowledge that project preregistration is preferrable but we hope that registration with a public registry at this point allows for transparency in the interpretation and analysis of the results.
