# Supplementary material for: Exploring perspectives on implementing cervical length screening to prevent preterm birth in nulliparous women: protocol for the PRE-EMPT qualitative work packages
Source: BMJ Open. 2026 Oct 7;16(10):e121899. doi: 10.1136/bmjopen-2026-121899 (PMC13646949; doi:10.1136/bmjopen-2026-121899)
Supplement: Supplementary file 2 [file bmjopen-16-10-s001.docx]

**Supplemental material**

**Examples of interview guides**

**Work package 1: Healthcare professional**

**HCP**

**Familiarity and knowledge**

What is your profession (e.g. obstetrician, midwife, sonographer, operational team)?

Do you have any management responsibilities and/or influence on implementing new practices?

- Can you think of examples of new practices you have been involved in?

What experience do you have of cervical length screening?

- Could you describe how do you do cervical length screening e.g. do you do abdominal scan first and then CLS? What do you think is the best way of conducting cervical length scanning?
- Do you use a pillow or a wedge to help you conduct cervical length scanning [to put under the patient’s bottom to be able to insert the probe]?

Do you have access to vaginal probes when you need them?

Could you describe the cleaning protocol for used probes in your department? *E.g. UV units, chemical solutions…*

In term of probe covers, how do you deal with women who have a latex allergy? What probe covers do you use?

Do you think cervical length scanning is effective in preventing pre-term birth?

What do you think would be the potential benefits and risks of introducing cervical length screening for low-risk nulliparous women?

- Do you think it is likely incidental findings will be noted? *E.g. cysts, polyps, FGM, sexual abuse…*
- If so, what sort of findings would you consider significant enough to inform the woman and/or refer to additional care?
- Do you feel confident and supported discussing these incidental findings with women and their partners? If you do not feel supported to discuss these findings, what resources you may need to feel supported? *E.g. more training, information sheet suggesting on how to discuss the findings, referral pathway…*

Do you think progesterone is effective in preventing pre-term birth?

- Has it ever happened to you that a patient declined the offer of progesterone?

What would motivate you to adopt this practice? What might affect your willingness?

**Skills and knowledge**

*If experienced:* Have you received any formal training or professional development on cervical length screening?

- Which aspects were helpful and which could be improved?

What skills or knowledge do you feel you would need to effectively implement this intervention?

- *If no experience:* What would it take for you to feel confident undertaking a cervical length scan?

The PRE-EMPT study will quality assure all cervical length scans and offer support to anyone who would benefit from it.

- How would you feel about this?
- How could this element of the study be delivered most effectively?

How would you like to receive feedback [on submitted scan images]? Individually or as part of a group?

**Team dynamics**

How do you typically collaborate with other healthcare providers (e.g., obstetricians, midwives, sonographers, operational team) when making decisions about pre-term birth prevention?

- Are there professional groups who are typically engaged with less?

To the best of your knowledge, how do your colleagues feel about the use of cervical length screening in practice?

Are there any team or institutional norms that would either support or hinder the adoption of this specific practice?

Can you think of any other interventions which have been implemented in your service? Were these effective? What were the barriers and facilitators to implementation?

- How did the culture in your team/service affect the implementation?

**Patients**

What do you expect the reactions from women to be to cervical length screening?

- Have you ever had women refuse a cervical length scan? What are your reflections on that experience?

How do you discuss the possibility of cervical length screening / pre-term birth prevention with your patients?

- What would enable you to feel confident having these conversations with patients?
- Would you feel comfortable telling a patient if you identified a short cervix?
- Would it be useful to have a sheet suggesting how to discuss risks of pre-term labour with patients?

How do you currently manage language barriers in care?

- Would you want anything to be different for cervical length scans?

**Institutional support and resources**

What resources do you think would be or are necessary for implementing routine cervical length screening?

The PRE-EMPT study is funding an additional 15 minutes per appointment for a cervical length scan. Do you think this will be sufficient?

*If not covered:*

Can you perceive any workforce problems?

- Is there a regular supply of/process of using bank sonographers in your service?

Can you perceive any problems with a lack of equipment?

Can you perceive any problems with a lack of space?

Do you perceive that the need for chaperones might be a barrier?

- Do you typically offer a chaperone for a transvaginal scan?
- Which staff members act as chaperones? Does this pose any problems to the delivery of scan clinics right now?

How does your hospital already support initiatives aimed at reducing pre-term birth?

- What were the facilitators and barriers to implementing these initiatives?

If sonographer:

Could you describe how patient lists are designed in your Trust? Who is responsible for designing them and how ling in advance are these lists designed?

**Policies and procedures**

Are you aware of any institutional policies or guidelines related to cervical length screening or pre-term birth prevention?

To the best of your knowledge, how do you think leadership at your institution would support or oppose the introduction of this intervention?

**Wider cultural and contextual factors**

What are the factors beyond your specific hospital that might make it easy or hard to implement cervical length screening?

- Politics
- Finances
- Workforce
- Scrutiny of maternity services
- Cultural attitudes towards women /pre-term birth

In your view, how does the wider culture and political environment in maternity healthcare influence the availability and implementation of interventions like cervical length screening?

**Summary**

Finally, reflecting on your answers, what do you perceive as the main facilitators and barriers to implementing cervical length screening for low-risk nulliparous women in your practice or institution?

Is there anything else it would be helpful for us to know?

Is there anyone you know who you think it would be good for us to interview?

The PRE-EMPT study is looking into the feasibility and efficacy of introducing cervical length screening. What evidence do you think we need to produce to be able to show that it is a worthwhile intervention to roll out nationally?

*What data do you collect as part of your preterm clinic?

**Work package 2: Women**

Suggested Topic guide for WP2

Exploring patient acceptability towards receiving a scan that could help prevent pre-term birth.

Semi-structured topic guide – example questions to guide discussion

*Capability*

*Focus: Understanding knowledge, skills, and abilities regarding the scan.*

*Psychological Capability*

- How much do you understand about the purpose of the scan in preventing pre-term birth?
- Do you feel you have enough information to make an informed decision about receiving the scan? [probe for psychological impact – ask if their anticipated feelings would inform their choice]
- Are there any gaps in your knowledge that might prevent you from opting for the scan?

*Physical Capability*

- Are you physically able to attend appointments for the scan?
- What challenges might you face in physically accessing this service?

*Opportunity*

*Focus: Environmental and social factors that influence access to the scan.*

*Physical Opportunity*

- Are there any barriers in accessing the location where the scan is provided (e.g., distance, transportation)?
- How convenient is it for you to schedule and attend the scan within your routine?
- Do you feel the healthcare system is supportive in providing this service?

*Social Opportunity*

- Have you discussed the scan with your midwife/obstetrician? If so, how did that conversation influence your decision?
- Do you have support from family or friends in deciding whether to receive the scan?
- How do you think the broader community views this scan? Is it commonly recommended or discussed?

*Motivation*

*Focus: The drive to undergo the scan, including beliefs and emotions.*

*Reflective Motivation*

- Do you believe that receiving the scan will help reduce your risk of pre-term birth?
- What are the main reasons you would (or would not) choose to receive the scan?
- Have you received any advice or recommendations from healthcare professionals regarding the scan?

*Automatic Motivation*

- How do you feel emotionally about receiving a scan? (e.g., fear, reassurance, anxiety) [probe for emotional impact – ask if their anticipated feelings would inform their choice]
- Are there any habits or routines in your life that would make it easier or harder to follow through with attending the scan?

*Finally…*

- Do you think your intentions towards your future care or health would change because of the scan?
- What do you think would make receiving the scan easier for you or others in similar situations?
- Do you have any concerns or suggestions regarding the healthcare system’s role in offering this scan to prevent pre-term birth?
- Do you have any other comments?

**Thank and close**
